# Compartmentalized, functional role of angiogenin during spotted fever group rickettsia-induced endothelial barrier dysfunction: evidence of possible mediation by host tRNA-derived small noncoding RNAs

**DOI:** 10.1186/1471-2334-13-285

**Published:** 2013-06-23

**Authors:** Bin Gong, Yong Sun Lee, Inhan Lee, Thomas R Shelite, Nawapol Kunkeaw, Guang Xu, Kwanbok Lee, Sung Ho Jeon, Betty H Johnson, Qing Chang, Tuha Ha, Nicole L Mendell, Xiaodong Cheng, Donald H Bouyer, Paul J Boor, Thomas G Ksiazek, David H Walker

**Affiliations:** 1 Department of Pathology, University of Texas Medical Branch at Galveston, 301 University Boulevard, Galveston, Texas, USA; 2 Center for Biodefense and Emerging Infectious Diseases, University of Texas Medical Branch at Galveston, Galveston, Texas, USA; 3 Department of Biochemistry and Molecular Biology, University of Texas Medical Branch at Galveston, 301 University Boulevard, Galveston, Texas, USA; 4, miRcores, Ann Arbor, Michigan, USA; 5 Department of Pharmacology and Toxicology, University of Texas Medical Branch at Galveston, Galveston, Texas, USA

## Abstract

**Background:**

Microvascular endothelial barrier dysfunction is the central enigma in spotted fever group (SFG) rickettsioses. Angiogenin (ANG) is one of the earliest identified angiogenic factors, of which some are relevant to the phosphorylation of VE-cadherins that serve as endothelial adherens proteins. Although exogenous ANG is known to translocate into the nucleus of growing endothelial cells (ECs) where it plays a functional role, nuclear ANG is not detected in quiescent ECs. Besides its nuclear role, ANG is thought to play a cytoplasmic role, owing to its RNase activity that cleaves tRNA to produce small RNAs. Recently, such tRNA-derived RNA fragments (tRFs) have been shown to be induced under stress conditions. All these observations raise an intriguing hypothesis about a novel cytoplasmic role of ANG, which is induced upon infection with *Rickettsia* and generates tRFs that may play roles in SFG rickettsioses.

**Methods:**

C3H/HeN mice were infected intravenously with a sublethal dose of *R. conorii*. At days 1, 3, and 5 post infection (p.i.), liver, lung and brain were collected for immunofluorescence (IF) studies of *R. conorii* and angiogenin (ANG). Human umbilical vein endothelial cells (HUVECs) were infected with *R. conorii* for 24, 48, and 72 hrs before incubation with 1μg/ml recombinant human ANG (rANG) in normal medium for 2 hrs. HUVEC samples were subjected to IF, exogenous ANG translocation, endothelial permeability, and immunoprecipitation phosphorylation assays. To identify small non-coding RNAs (sncRNAs) upon rickettsial infection, RNAs from pulverized mouse lung tissues and HUVECs were subjected to library preparation and deep sequencing analysis using an Illumina 2000 instrument. Identified sncRNAs were confirmed by Northern hybridization, and their target mRNAs were predicted *in silico* using BLAST and RNA hybrid programs.

**Results:**

In the present study, we have demonstrated endothelial up-regulation of ANG, co-localized with SFG rickettsial infection *in vivo*. We also have provided direct evidence that rickettsial infection sensitizes human ECs to the translocation of exogenous ANG in a compartmentalized pattern at different times post-infection. Typically, exogenous ANG translocates into the nucleus at 24 hrs and to the cytoplasm at 72 hrs post-infection. The ANG cytoplasmic translocation enhances phosphorylation and destabilization of VE-cadherin and attenuates endothelial barrier function. Of note, deep sequencing analysis detected tRFs, mostly derived from the 5'-halves of host tRNAs, that are induced by ANG. Northern hybridization validates the two most abundantly cloned tRFs derived from tRNA-ValGTG and tRNA-GlyGCC, in both mouse tissues and human cells. Bioinformatics analysis predicted that these tRFs may interact with transcripts associated with the endothelial barrier, the host cell inflammatory response, and autophagy.

**Conclusions:**

Our data provide new insight into the role of compartmentalized ANG during SFG rickettsioses, and highlight its possible mediation through tRFs.

## Background

Spotted fever group (SFG) rickettsioses are of global public health importance [1-4]. These tick-borne diseases are caused by obligately intracellular bacteria of the genus *Rickettsia* (*R.*) that include *R. rickettsii* (causative agent of Rocky Mountain spotted fever [RMSF]) and *R. conorii* (causative agent of Mediterranean spotted fever) [5,6]. Up to 20% of untreated and 5% of treated RMSF cases result in a fatal outcome caused by systemic microvascular hyperpermeability [2]. *R. rickettsii* is also designated as a potential bioterror agent [7].

Spotted fever group rickettsiae target host endothelial cells that line the blood vessels. Microvascular endothelial barrier dysfunction is the central enigma in SFG rickettsioses [1-4,8]. Many possibilities have been suggested to explain the cause of endothelial permeability. However, a defined mechanism has not been determined for this phenomenon [9,10]. The endothelial barrier includes cell–cell adhesion proteins at cell junctions. Our recent study indicated that upon infection by SFG rickettsiae, phosphorylation of adhesion proteins directly attenuates homophilic protein–protein interactions at the endothelial adherens junctions [11], but the underlying molecular mechanism remains unclear.

Previous studies suggested that a subset of angiogenic factors had inconsistent effects on the regulation of endothelial barrier function [12-16]. Human angiogenin (ANG) is one of the earliest identified angiogenic factors, produced mainly by hepatocytes, and correlates with not only angiogenesis but also vascular and tissue homeostasis in the human placenta [17-19]. Endogenous ANG accumulation in endothelial nuclei is a general requirement for endothelial proliferation and angiogenesis induced by other angiogenic factors including VEGF, FGF, and EGF [20]. Moreover, exogenously added ANG rapidly translocates into the nucleus of growing endothelial cells, binds to DNA, activates rRNA transcription, and promotes protein synthesis [21-23]. Endocytosis of ANG is mediated by an unidentified membrane receptor and can be inhibited by neomycin [24]. The relocalization of ANG is independent of lysosome and the microtubule system, but is strictly dependent on endothelial cell density [21,25]. No nuclear ANG is detected in confluent quiescent endothelial cells [20,21]. Furthermore, ANG does not induce any detectable cellular internalization in primary non-blood vessel cells (including epithelial cells and fibroblasts) regardless of the cell density, except immortalized cell lines [21,22].

Given the fact that ANG interacts with ANG-specific binding DNA sequences in endothelial cells to exhibit a wide range of cellular responses to other angiogenic factors including migration, proliferation, and tube formation [18,20], we undertook to determine whether ANG is also involved in regulating endothelial barrier stability. Furthermore, we sought to determine whether the endocytosis of ANG can be activated if confluent endothelial cells are infected with SFG rickettsiae, inducing bioactivity to regulate endothelial permeability. This would be the first study to address this issue.

Recent studies on biological activity of ANG have extended their function from enabling cell growth and proliferation to sustaining endothelial cell survival under various stresses including heat shock, hypothermia, hypoxia, and radiation [26-30]. These emerging roles of ANG are distinct from those of nuclear-located ANG and are envisioned to be relevant to cytoplasmic-localized ANG, causing specific cleavage of tRNA, processed by its unique capacity as a tRNA-specific ribonuclease (RNase) [18,31]. However, the referenced studies did not show concomitant enhanced expression of ANG in cytoplasmic compartments under these adverse conditions [18].

Besides the widely-studied microRNAs, novel types of small non-coding RNAs (sncRNAs) have been (and are being) discovered by virtue of recent innovations in deep sequencing (meaning ultra-high throughput sequencing or next generation sequencing) [32-34]. Among them, sncRNAs derived from tRNA cleavage have gained significant attention recently [35-37]. Although once regarded as junk RNA, these RNAs are generated by a specific cleavage of mature or precursor tRNA and are termed tRFs (tRNA-derived RNA fragments) [36]. By definition, tRFs are precisely aligned with mature or precursor tRNA at either end and are distinguished from non-tRFs that are randomly mapped to anywhere in tRNA. tRFs can be further sub-grouped into tRF-5 and -3 series [36], depending on which end they contain.

Several groups have observed stress-induced tRNA cleavage[26-29,38], which generates tRFs. Recently, most tRFs were captured by deep sequencing in diverse organisms ranging from yeasts to humans [29,36-40]. tRFs are thought to be generated by a single endonuclease cleavage of tRNA [36]. In general, tRF-5 series have been more commonly detected than tRF-3 series. Several reports [26,28,29] have indicated that tRF-5 series are generated by ANG and have been characterized to have a functional pathobiological role in several cell models [41].

ANG belongs to the RNase A superfamily and is known as the only angiogenic protein to specifically cleave tRNA *in vivo* and *in vitro*[29,42,43]. tRNA cleavage is speculated to occur in the cytoplasm, especially when ANG gains access to cytosolic tRNAs during stress [28,31]. Thus ANG’s function in the cytoplasm is distinct from its regulation of rRNA transcription in the nucleus [20,44]. However, there is no direct evidence of cytoplasmic translocation of ANG in endothelial cells triggered by adverse events [18].

Here, we hypothesize that infection with SFG *R. conorii* initiates translocation of exogenous ANG into different endothelial cellular compartments at different times post-infection, leading to distinct functional consequences mediated by cytoplasmic ANG-induced tRNA-derived sncRNAs. In the present study, we have obtained direct evidence to demonstrate that rickettsial infection initiates compartmentalized translocation of exogenous ANG in confluent human primary endothelial cells. By investigating the function and potential effects of this compartmentalized translocation of ANG, we found that rickettisial infection-triggered cytoplasmic translocation of ANG, enhanced phosphorylation of VE-cadherin, reduced VE-cadherin stability, and attenuated endothelial barrier function. Furthermore, high throughput deep sequencing analysis and Northern hybridization validation revealed novel tRNA-derived sncRNAs that are predicted by *in silico* analysis to interact with endothelial functions.

## Methods

### Ethics statement

All experiments and procedures with animals were conducted according to the National Institutes of Health guidelines for housing and care of laboratory animals and performed under the protocol (IACUC 9007082), which was reviewed and approved by the Institutional Animal Care and Use Committee and the Institutional Biosafety Committee at the University of Texas Medical Branch, Galveston.

### Mice

Female 8-12 week old C3H/HeN mice were purchased from the Jackson Laboratory (Bar Harbor, ME). Experimentally infected mice were housed in a standard CDC-approved biosafety level 3 facility.

### Reagents

Recombinant human ANG (rANG) was purchased from R&D Systems (Minneapolis, MN). Cell culture medium Prigrow I and fetal bovine serum were obtained from Applied Biological Materials (Richmond, BC, Canada). Unless otherwise indicated, all reagents were purchased from ThermoFisher Scientific Inc (Waltham, MA).

### *Rickettsia* purification

*R. conorii* (strain Malish 7) was obtained from the American Type Culture Collection. A 10% yolk sac suspension of *R. conorii* from infected embryonated chicken eggs diluted in sucrose-phosphate-glutamate (SPG) buffer (0.218 M sucrose, 3.8 mM KH_2_PO_4_, 7.2 mM K_2_HPO_4_, 4.9 mM monosodium l-glutamic acid, pH 7.0) was propagated through two passages in Vero cells [11]. *R. conorii* were isolated using a bead-based protocol as described previously (11). Purified rickettsiae were frozen in SPG buffer at -80°C. Rickettial content of the frozen stocks was determined by plaque assay and TCID_50_ assays on Vero cells, and yielded approximately 1 × 10^9^ bacterial cells per ml. Uninfected Vero cells were processed by the same procedure as control material.

### *In vivo* animal experiment

After dilution in PBS, a sublethal dose of *R. conorii* (2×10^5^ plaque-forming units in 200 μl per mouse) was inoculated intravenously via the tail vein. Control (mock infected) mice were inoculated with 200 μl of processed uninfected Vero cells in PBS. At days 1, 3, and 5 post-infection (p.i.), liver, lung and brain were collected and immersion fixed in methanol for 72 hours at 4°C prior to further processing for RNA sample preparation. For immunofluorescence (IF) studies of *R. conorii* and ANG, all tissues were immersion fixed in 10% neutral-buffered formalin for 24 hrs prior to being transferred to 70% ethanol at 4°C until processing.

### Cell culture

HUVECs (Cell application, Atlanta) were cultivated in Prigrow I medium supplemented with 10% heat-inactivated fetal bovine serum in 5% CO_2_ at 37°C. All experiments were performed between passages 5 and 7, and cells were maintained in Prigrow I medium with 3% fetal bovine serum.

For the ANG translocation studies and IF assays, HUVECs were cultured on round glass coverslips (12 mm diameter, Ted Pella, Redding, CA) until they were 90% confluent. The cells were then infected with *R. conorii* at a multiplicity of infection (MOI) of 10. After 24, 48, and 72 hrs, the cells on the coverslips were washed three times in phosphate-buffered saline (PBS) before the downstream studies were performed.

### Immunofluorescence

Cells were fixed with cold methanol at 24, 48, or 72 hrs post-infection. Each experiment was repeated three times. The primary antibodies, a mouse monoclonal IgG against ANG (1:500) (Clone 14017.7, Thermo Scientific, Rockford, IL), a mouse monoclonal IgG against VE-cadherin (1:500) (Clone TEA1/31, Meridian Life Science, Saco, ME), and a rabbit polyclonal IgG antibody against SFG rickettsiae (1:2000), were added and incubated for 2 hrs. For IF studies on mouse tissue samples, deparaffinized and rehydrated sections were blocked with unconjugated AffiniPure Fab fragment goat anti-mouse IgG (H+L) (Jackson ImmunoResearch Labs, Code Number: 115-007-003) for 1 hour at room temperature before incubation with mouse monoclonal IgG against ANG (1:200) and rabbit polyclonal IgG antibody against SFG rickettsiae (1:1000) overnight at 4°C. The reactivities of antibodies to ANG and VE-cadherin in human and mouse samples have been confirmed by the manufacturers. ANG or VE-cadherin and rickettsiae were detected with secondary goat anti-mouse Alexa 488 and goat anti-rabbit Alexa 594 conjugated antibodies (Invitrogen, Carlsbad, CA), respectively. Nucleic were counter-stained with Prolong Gold antifade reagent with DAPI (Invitrogen, Carlsbad, CA). A mouse monoclonal IgG1 (Thermo Fisher Scientific, Fremont, CA) or Rabbit Polyclonal IgG (Thermo Fisher Scientific, Fremont, CA) was served as a negative control at same working concentration as all above primary antibodies respectively to assure specific staining detection. Fluorescent images were taken and analyzed using an epifluorescence or confocal microscope.

### Exogenous ANG translocation assay

HUVECs were seeded at a density of 5× 10^3^ cells per cm^2^ on a coverslip and infected with *R. conorii* at an MOI of 10. At different times post-infection, they were washed with serum-free medium and incubated with 1 μg/mL rANG in medium and incubated at 37°C. Previous studies from other groups exposed HUVECs to rANG for 1 hr, resulting in significant nuclear translocation and increased rRNA transcription [20,21]. In the present study, we sought to demonstrate that ANG’s distinct compartmentalized function correlated with its different biochemical effects on rRNAs and tRNAs. A preliminary experiment using recombinant human ANG to lyse human total tRNA *in vitro* revealed that a 2-hour reaction produced a significant quantity of tRNA-derived fragments (see Additional file 1). Therefore, we utilized a two-hour incubation at a dose of rANG of approximately 12.5 pg per cell. Cells were washed with PBS thrice, fixed with methanol at -20°F, and washed again with PBS containing 30 mg/mL of bovine serum albumin. The fixed cells were subjected to the IF procedure as above.

### Endothelial cell permeability assay

The permeability of HUVECs upon infection of *R. conorii* at a MOI of 10 was determined using an *in vitro* vascular permeability assay (Millipore, Billerica, MA) as previous described [11]. Briefly, HUVECs were seeded onto type I rat-tail collagen-coated polycarbonate Transwell filters (6.5-mm diameter and 3-μm pore size; Millipore, Billerica, MA), and confluent monolayers were inoculated with *R. conorii* or mock-infected control Vero cell material processed using a bead-isolating protocol. At different times post-infection, HUVECs were exposed to ANG (1μg/ml) or PBS for two hrs before permeability was assessed by adding 0.5 mg/ml of fluorescein isothiocyanate (FITC)-dextran (Sigma, St. Louis, MO) to the top chamber. After 1 hr, FITC-dextran present in the bottom compartment was assayed using a BioTek Synergy 2 multi-mode microplate reader (485 nm excitation, 530 nm emission). The fold-change in fluorescence intensity over the basal permeability of monolayers was used as an indicator of paracellular permeability of assessed monolayers. Experiments were performed in replicates of four.

### Immunoprecipitation (IP) phosphorylation assay

To study VE-cadherin phosphorylation, cell lysates were prepared with an ice-cold RIPA lysis buffer (Santa Cruz Biotechnology, Santa Cruz, CA). After centrifugation at 12,000×*g* for 20 min, the protein supernatant was collected. Equal amounts of protein with optimal Dynabead Protein G (Invitrogen, Carlsbad, CA) conjugated with anti-VE-cadherin antibody were incubated for 2 hr at room temperature. The Dynabead-antibody-antigen complex pellets were precipitated and separated using DynaMag-2 (Invitrogen, Carlsbad, CA). The pellet was washed three times with PBS, and resuspended in 20 μl of SDS sample buffer (Invitrogen, Carlsbad, CA) and heated for 10 min at 70°C. Samples were then separated by gel electrophoresis followed by immunoblotting. A mouse monoclonal anti-phosphotyrosine antibody, 4G10 (Millipore, Billerica, MA) was used at dilution of 1:500 for detection of proteins containing phosphotyrosine. All experiments were performed in replicates of three.

### *In vitro* ANG lyse human tRNA

The 10 μl of reaction mixture contained 250 ng of purified human total tRNA (Bio S&T, Montreal, Quebec, Canada) and rANG (1μg/ml) in reaction buffer (30 mM HEPES pH 7.4, 30 mM NaCl, and 0.01% bovine serum albumin). The reaction was performed at 37°C for the indicated times and quenched by adding 10 μl of Gel Loading Buffer II (Life Technologies Corp.). Cleavage products were resolved in 15% denaturing polyacrylamide gel with 7 M urea and visualized by ethidium bromide staining.

### RNA extraction and deep sequencing

From the *in vivo* study, methanol fixed mouse lung tissues were homogenized using a pulverizer (Spectrum Laboratories, Rancho Dominiquez, CA) in an RNase-free environment prior to the total RNA extraction procedure. Briefly, approximately 100 mg of chopped lung tissue in the pulverizer device were snap-frozen in liquid nitrogen for 5 minutes prior to pulverization. Pulverized tissue powder was blended in Trizol reagent (Invitrogen) at 4°C for further RNA preparation. From the *in vitro* HUVECs experiments, methanol fixed cells were collected at 24, 48, or 72 hr p.i., for preparation of total cellular RNA using Trizol reagent.

The steps involved in library preparation were (1) size fractionation of sncRNAs from total RNA; (2) adaptor ligation (to provide primer binding sites for subsequent steps); (3) cDNA synthesis; (4) PCR-amplification (one primer contained index sequences to be read during sequencing); (5) deep sequencing analysis using an Illumina 2000 (Illuminar, San Diego, CA) for. High throughput deep sequencing studies were performed in the Molecular Genomics Core at UTMB.

### RNA mapping and expression confirmation of tRFs by Northern blot

After deep sequencing, a total of 70,544,933 sequence reads was generated from mock and infected samples. After adaptor sequence removal, sequences whose read numbers were less than 10 were discarded. Sequences (cloned ≥ 10) were identified by mapping to several sequence databases. Small RNAs were mapped using Novoalign software (Novocraft Technologies, Selangor, Malaysia) allowing two mismatches. After initial alignment, further processing was performed using in-house programs and SAMtools [41]. First, uniquely aligned reads and sequences aligned to more than one genome location (ambiguously aligned reads) were separated. The ambiguously aligned reads were then randomly assigned to one location and combined with uniquely aligned reads for the downstream analyses.

**Figure 1:**
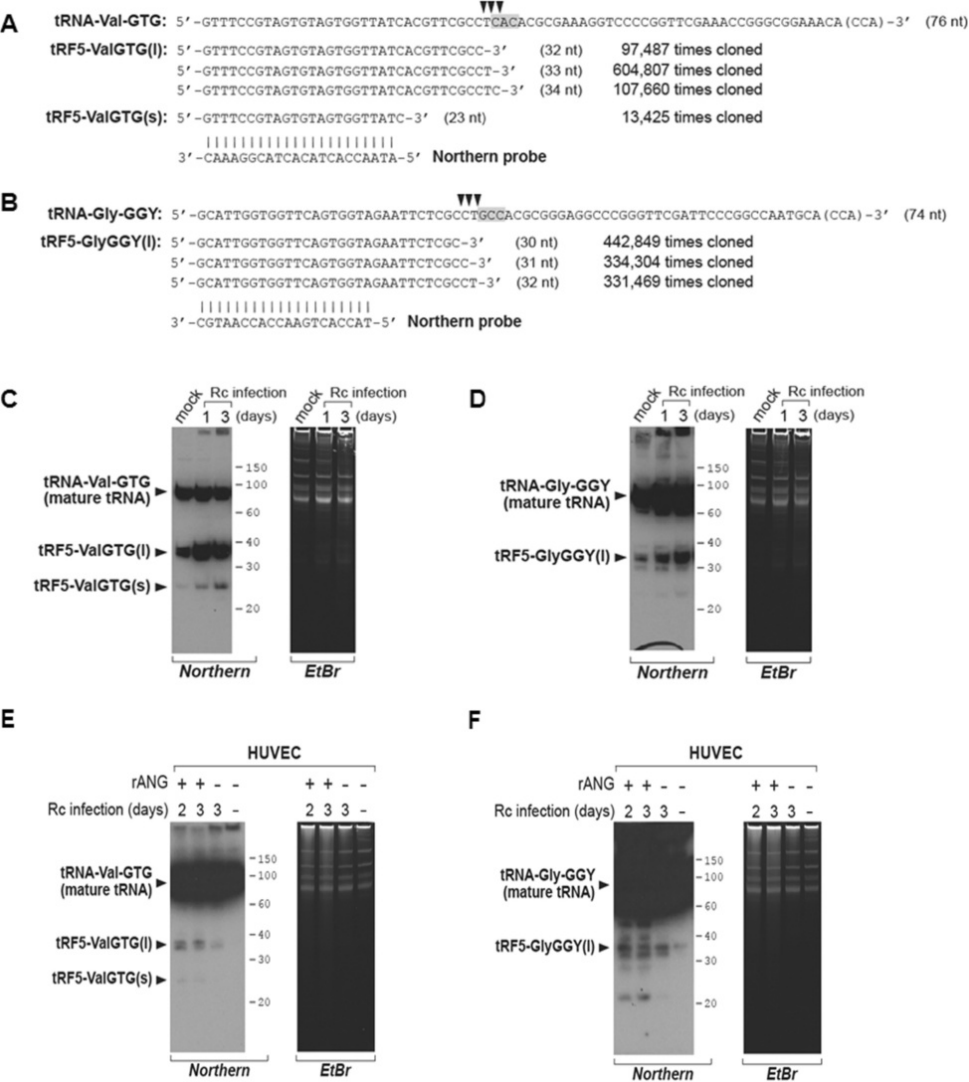
**Experimental validation of tRF expression. A** and **B**. Sequence alignment of tRF-5s with their parental mature tRNAs. The most abundantly cloned tRF-5 isoforms, together with their cloning numbers, are shown. For tRF5-ValGTG, our deep sequencing detected a significant quantity of longer isoforms (designated by “tRF5-ValGTG(l)”) and a short isoform (designated by “tRF5-ValGTG(s)”), both of which were detected in our Northern hybridization (panels shown below). In mature tRNAs, arrowheads on the top indicate cleavage sites based on the isoforms. Anticodons are highlighted by grey. “CCA” in parenthesis indicates a CCA sequence that is post-transcriptionally added to the 3'-end of tRNA. The lengths of each tRNA and tRF-5s are indicated in parentheses. Northern probes (used in next panels) are also aligned and shown. **C**-**F**. Northern hybridization of each tRF-5. Photographs of an autoradiogram (designated “Northern” at the bottom) and the ethidium bromide-stained gel (designated “EtBr” at the bottom) are shown. Molecular size markers (in nts) and identities of each band are also indicated. Northern experiments were performed on the mouse tissues used in the deep sequencing (panels **C**-**D**) and HUVECs after indicated treatments (panels **E**-**F**).

Northern hybridizations for sncRNAs were performed as described [36,41], with probes shown in Figure 1A-B. Briefly, total RNA was separated in a 15% denaturing polyacrylamide gel with 7 M urea, and then transferred to a positively charged nylon membrane (Amersham Biosciences, Piscataway, NJ). The membrane was hybridized with ^32^P-labeled probes in ULTRAhyb-Oligo solution (Life Technologies, Grand Island, NY), followed by washing according to the manufacturer's instructions.

### Computational prediction of target mRNAs

tRF5-ValGTG and tRF5-GlyGCC sequences were subjected to BLAST (Basic Local Alignment Search Tool) against the “human genome + transcript” with relaxed search parameters for initial screening. Specific parameters were: searching “somewhat similar sequences”, word size = 16, and expect threshold = 1000. Other parameters were automatically adjusted for short input sequences. The BLAST hits were further analyzed by using RNAhybrid. The binding energy between tRF and target was used to make a short list of targets whose Δ G value was <30 kcal/mol.

### Statistical analysis

Values are reported as mean ± SD. The data were analyzed using One Way ANOVA analysis (Sigmaplot, Sigma Stat, Jandel Scientific Software, San Rafael, CA). Statistical significance was considered as *P*<0.05.

## Results

### Upregulation of ANG in vascular endothelial cells after SFG rickettsial infection *in vivo*

**Figure 2:**
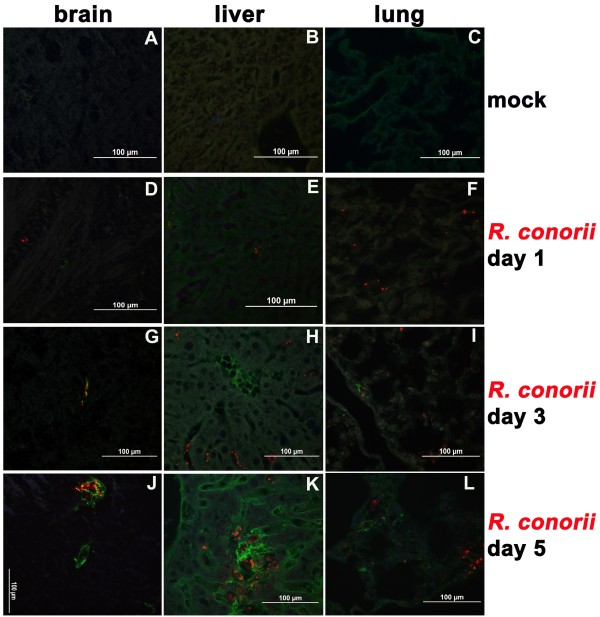
**Immunohistofluorescence studies show up-regulation of ANG in microvascular endothelial layers, co-localized with SFG rickettsiae in lesions, in mouse brains, livers, and lungs on days 3 and 5 post-infection.** Dual immunofluorescence staining of SFG rickettsiae (red) and ANG (green) in mouse tissues using a dual wave length filter system revealed that the ANG signal was restricted to microvascular endothelial layers in multiple organs or hepatocytes (green signals in images **A**-**F**). First appearing day 3 post-infection, compared to mock controls, *R. conorii* infection (2 × 10^5^ PFU, red signal) resulted in increased signal of ANG (green signals in image **G**-**L**) in the microvascular endothelial layers in brain, liver and lung. Up-regulated ANG is co-localized with *R. conorii* (red signal) in lesions on day 5 post-infection (image **J**-**L**). Nuclei of mouse cells are counter-stained with DAPI (blue).

ANG has been found to be expressed in a wide range of tissues, with the liver the major source for circulating ANG in plasma [17]. ANG has been implicated in the regulation of the cellular response to stress [18]. Upon infection with rickettsiae, ANG was induced, as visualized by IF (Figure 2).

Basal-level expression of ANG was detected sporadically in mock control and day 1 p.i. of 2 × 10^5^ plaque-forming units (PFU) of *R conorii*. The ANG signal was restricted in microvascular endothelial layers in multiple organs or hepatocytes (green signals in images A-F in Figure 2). On day 3 p.i., intense ANG signal was detected in vascular endothelial layers, co-localized with rickettsial signal in brain and rickettsial infection lesion in liver and lung (red signals in images G-I, Figure 2). In liver, enhanced accumulation of ANG signal was detected in endothelial layers of hepatic sinusoid blood vessels (Figure 2H), compared to hepatocytes. On day 5 p.i., ANG signal was more intense and restricted in blood-brain and blood-air barriers. Again, ANG and rickettsiae signals were co-localized (images J-L in Figure 2). In liver, compared to the endothelial layer of hepatic sinusoid blood vessel, there was stronger signal of ANG in rickettsial infectious lesions (Figure 2K).

### Rickettsial infection initiated compartmentalized translocation of exogenous ANG in endothelial cells

Accumulation of endogenous ANG in endothelial nuclei is a general requirement for endothelial proliferation and angiogenesis triggered by other exogenous angiogenic factors including VEGF, FGF, and EGF [20,21]. To obtain data to support functional studies in a human primary endothelial cell model, we determined the expression pattern of ANG in HUVECs following rickettsial infection using an IF-based exogenous ANG translocation assay [20,21].

**Figure 3:**
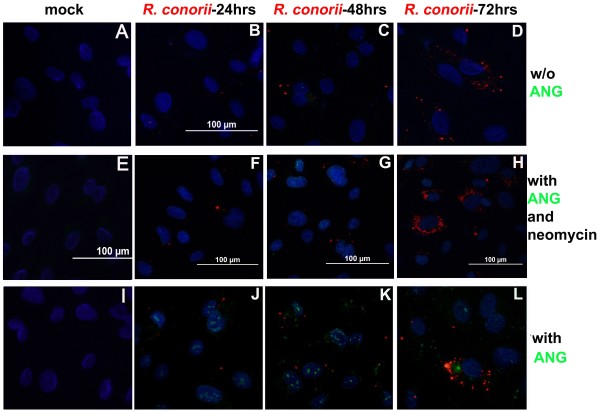
**SFG rickettsial infection initiated compartmentalized translocation of exogenous rANG in human primary endothelial cells.** Dual immunofluorescence staining of SFG rickettsiae (red) and ANG (green) in human umbilical vein endothelial cells (HUVECs) using a dual wave lengths filter system revealed that there was no significant detectable endogenous ANG in endothelial cells (images **A**-**D**). *R. conorii* infection triggered compartmentalized translocation of exogenous rANG at different times p.i. (images **J**-**L**). Neomycin reduced cellular internalization of exogenous rANG in SFG rickettsiae-infected endothelial cells (images **F**-**H**). Nuclei of HUVECs are counter-stained with DAPI (blue).

First, infection by *R. conorii* induced no significant signal of detectable endogenous ANG in endothelial cells, in either nuclear or cytoplasmic locations (Figure 3A-D). However, *in vivo* observation (Figure 2) revealed a dynamic up-regulation of ANG in microvascular endothelial cells following SFG rickettsial infection, displaying an organ-specific expression pattern. Previous studies also indicated that exogenous ANG underwent nuclear translocation in proliferative endothelial cells [21]. However, no nuclear ANG can be detected in confluent stable endothelial cells [20]. We tested whether infection with SFG rickettsiae can trigger endocytosis of exogenous ANG at 24, 48, or 72 hrs p.i.

In HUVECs exposed to ANG for 2 hours, IF studies revealed that SFG rickettsial infection could initiate endocytosis of exogenously added ANG in confluent quiescent HUVECs (Figure 3I-L). Furthermore, *R. conorii* infection initially triggered nuclear translocation of exogenous ANG at 24 hrs p.i. Most importantly, ANG was translocated into the cytoplasm at 48 hrs p.i. (Figure 3K) and predominated in the cytoplasmic location at 72 hrs p.i. (Figure 3L). Moreover, neomycin, an aminoglycoside antibiotic and also an inhibitor that blocks nuclear translocation of human ANG [24], caused reduced cellular internalization of human ANG in rickettsial-infected HUVECs (Figure 3E-H). Administration of neomycin at 10 μM for 2 hrs in culture medium before fixation did not affect the rickettsial load in endothelial cells (assessed by real time PCR, data not shown).

### Compartmentalized functional role of ANG on human endothelial barrier function after infection with SFG rickettsiae

**Figure 4:**
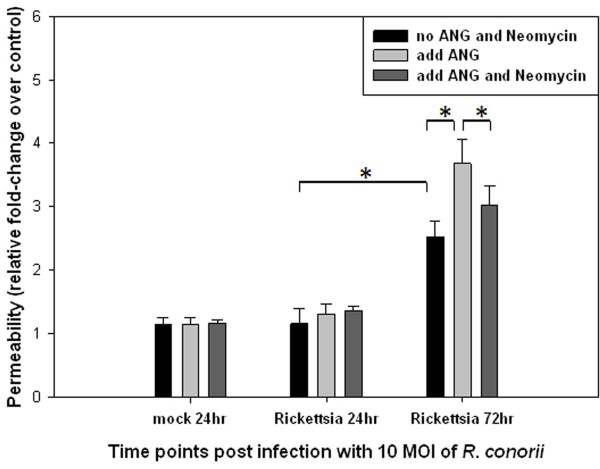
***conorii *infection-initiated cytoplasmic translocation of exogenous rANG and enhanced para-endothelial hyperpermeability at 72 hrs p.i., this effect can be attenuated by co-administration of neomycin with rANG.** Endothelial cells were seeded on type I rat-tail collagen-coated polycarbonate transwell filters and infected with *R. conorii* at an MOI of 10 in triplicate, or mock infected. After 24 and 72 hr, HUVECs were exposed to ANG or co-administration of neomycin with ANG for two hrs. FITC-dextran was added to the upper chamber medium, and the presence of FITC dextran in the lower chamber was quantified after 1 hr. The results are expressed as the fold-increase in monolayer permeability over basal permeability levels (* *P*< 0.05). Experiments were performed in replicates of four.

To determine if the SFG rickettsiae-triggered endocytosis of exogenous rANG is relevant to endothelial barrier dysfunction, we performed in vitro assays to measure vascular endothelial permeability. In HUVEC monolayers, R. conorii triggered cytoplasmic translocation of exogenous rANG (Figure 3L) and enhanced para-endothelial hyperpermeability at 72 hr p.i. (Figure 4). Such augmentation could be attenuated by coadministration of neomycin with rANG (Figure 4). This result is the first evidence supporting the concept that SFG rickettsiae-triggered endocytosis of exogenous ANG results in enhanced endothelial permeability.

### Cytoplasmic ANG enhances tyrosine phosphorylation of VE-cadherin and VE-cadherin internalization into endothelial cells

**Figure 5:**
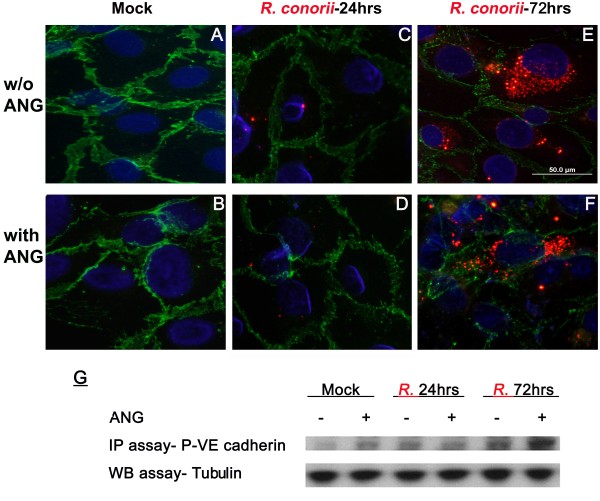
***R. conorii *infection-initiated cytoplasmic translocation of exogenous rANG enhanced VE-cadeherin internalization into endothelial cells and tyrosine phosphorylation of VE-cadherin at 72 hrs p.i.** Dual immunofluorescence staining of SFG rickettsiae (red) and VE-cadherin (green) in human umbilical vein endothelial cells (HUVECs) using a dual wave length filter system revealed that internalized VE-cadherin could be detected at 72 hrs p.i. (image **E**), compared to mock and 24 hrs p.i. (image **A**, **C**). Addition of rANG shows an enhancing effect on the internalization of VE-cadherins in HUVECs at 72 hrs p.i. (image **F**). A representative immunoprecipitation (IP) study suggested that exogenous rANG enhances phosphorylation of VE-cadherin (p-VE-cadherin) at 72 hrs p.i. (image **G**). Nuclei of HUVECs are counter-stained with DAPI (blue).

Our previous studies revealed that SFG rickettsiae induces microvascular hyperpermeability via phosphorylation of VE-cadherins [11]. IF imaging evidence from other groups [12,45] directly demonstrated that phosphorylation of VE-cadherins trigger internalization of VE-cadherin, decreasing endothelial barrier function [10]. In this study, internalized VE-cadherin was detected by immunofluorescent microscopy at 72 hrs p.i. (Figure 5E), compared to mock and 24 hrs p.i. (Figure 5A-D). Addition of ANG showed an enhancing effect on the internalization of VE-cadherins in HUVECs at 72 hrs p.i. (Figure 5F). An immunoprecipitation study revealed that exogenous ANG enhanced phosphorylation of VE-cadherin at 72 hrs p.i. (Figure 5G). There is no alteration in expression of total VE-cadherin between control and infected experimental monolayers at different time p.i. as detected by Western blot assay.

### Characterization of rickettsiae-triggered, ANG-induced, tRNA-derived sncRNAs

Recently, studies on the biological activity of ANG have been extended to sustaining endothelial cell survival in response to stress [26-29]. Such an emerging role of ANG correlates with its cytoplasmic localization, especially when ANG gains access to cytosolic tRNA during stress [28,31]. ANG is an RNase which cleaves tRNA [42], as also shown in the Additional Data file. When purified human tRNA was incubated with human recombinant ANG (hrANG), tRNA degradation was evident as seen by a decrease in the intensity of mature tRNA bands and an accumulation of smaller bands (Additional file 1).

Next, we determined whether the cytoplasmic ANG generated tRFs *in vivo*, by using deep sequencing. We obtained a total read of 70,544,933 from three experimental sets, each of which was replicated 3 times (Table 1). As expected, the most abundant class of sncRNAs was microRNAs that occupied approximately 60% of total reads, indicating that the RNA quality was preserved during the RNA isolation from mouse tissues. A similar fraction of microRNAs were cloned in previous deep sequencing experiments [36,41]. If non-specific RNA degradation occurred, the portion of microRNAs would have been much lower.

**Table 1:** Summary of high-throughput sequencing data

		**Mock**		**Rickettsia infection day 1**				**Rickettsia infection day 3**		
		Replicate 1	Replicate 2	Replicate 3	Replicate 1	Replicate 2	Replicate 3	Replicate 1	Replicate 2	Replicate 3
**Total reads**		7,976,072	7,579,606	7,072,909	9,086,972	9,303,189	8,233,584	7,000,534	7,478,639	6,813,408
**Mouse genome**		6,825,993	6,504,196	6,142,393	7,888,232	8,053,081	7,064,456	6,014,752	6,419,927	5,653,699
**(GRCm38/mm10)**										
**Mouse small RNAs**	microRNA	4,726,255	4,538,273	4,437,811	5,678,975	5,617,614	4,833,244	4,099,680	4,176,360	2,843,170
	piRNA	36,579	39,855	34,899	45,506	40,654	35,252	35,230	36,925	30,880
	snoRNA	104,905	111,394	111,173	107,850	160,003	130,500	142,705	126,827	108,898
	snRNA	11,381	12,387	11,513	11,055	14,467	12,857	18,941	19,080	15,470
	rRNA	19,666	24,792	28,487	25,276	26,243	28,507	28,403	25,919	29,887
	**tRNA**	**388,179**	**239,575**	**150,429**	**278,754**	**457,630**	**452,977**	**337,384**	**597,214**	**700,422**
**tRNA matches**	tRF-5	362,050	212,503	122,029	249,710	423,795	420,596	304,148	548,458	647,353
	t-RF-3	319	302	579	511	487	580	427	553	571
	non-tRF	25,810	26,770	27,821	28,524	33,384	31,801	32,809	48,203	52,498

The read numbers are summarized and tabulated. Sequences matching tRNAs were further sorted into tRF-5, -3, and non-tRFs, as defined in the text. Mouse genome (GRCm38/mm10) indicates *Mus musculus* genome assembled by the Genome Reference Consortium (GRCm38), UCSC version 10.

**Figure 6:**
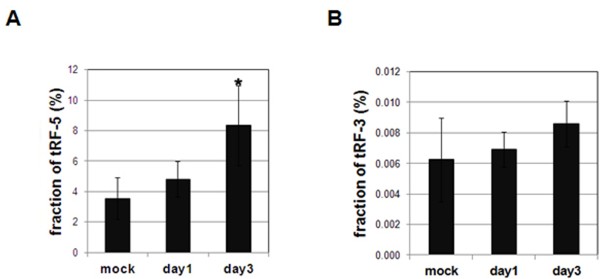
**ANG cleaves tRNA to generate tRF-5 series. A** and **B**. Fraction of tRF-5 (panel A) and tRF-3 (panel B) in the total small RNA population from mouse lungs. A relative cloning frequency of each tRF-5 (or -3) was calculated by normalizing the tRF’s read number to the total read number (as shown in Table 1). The normalized values are expressed in percentages. An average and a standard deviation were calculated from triplicate samples of each treatment.

tRFs, especially the tRF-5 series, are the second most abundant after microRNAs (Table 1). As described above, tRF-5 series are the 5'-side product derived from ANG cleavage. tRF-5’s domination (>90%) over tRF-3 and non-tRFs provided another piece of evidence that tRFs were not intermediates of random tRNA degradation. Most importantly, tRF-5s were significantly increased upon rickettsial infection (Figure 6A). For comparison, tRF-3s were barely captured and exhibited comparable cloning frequencies between mock- and rickettsiae-infected samples (Figure 6B). These data are in agreement with the re-localization of ANG into the endothelial cytoplasm upon SFG rickettsial infection.

Furthermore, we examined which particular tRF sequences were cloned in the deep sequencing. Based on read numbers, the five most abundantly cloned tRFs were sorted and tabulated in Table 2. Comparison of relative cloning frequencies (a tRF’s read number normalized to the sample’s read number) indicated that all of them were increased upon rickettsial infection. We chose the two most abundantly cloned tRFs for further inspection and experimental confirmation.

**Table 2:** The most abundantly cloned tRF-5s.

	**Sequence**	**Parental**	**Relative cloning frequency (‰)**		
			**Mock**	**Ricketsia infection**	
				**day 1**	**day 3**
tRF5-ValGTG	GTTTCCGTAGTGTAGTGGTTATCACGTTCGCCT*	tRNA-Val GTG, tRNA Val GTY	3.984	6.494	12.950
tRF5-GlyGCC	GCATTGGTGGTTCAGTGGTAGAATTCTCGC*	tRNA-Gly-GYY, tRNA-Gly-GGG	4.767	5.734	7.638
tRF5-GlyGCC (A to C)	GCCTTGGTGGTTCAGTGGTAGAATTCTCGC	tRNA-Gly-GYY, tRNA-Gly-GGG	2.977	3.954	4.791
tRF5-GluCTC	TCCCTGGTGGTCTAGTGGTTAGGATTCGGC	tRNA-Glu-GAG	0.700	0.501	1.108
tRF5-LysCTT	TCCCTGGTGGTCTAGTGGTTAGGATTCGGC	tRNA-Lys-AAG	0.429	0.577	1.144

For individual tRF-5 sequences, their relative cloning frequencies were calculated as described in the Figure 6 legend. Based on the value (expressed in ‰ [1 per 1,000]), the most abundantly cloned tRF-5s were selected and tabulated. Among the five tRF-5s, two (tRF5-ValGTG and tRF5-GlyGCC: highlighted by asterisks in their sequences) were chosen for further study. “tRF5-GlyGCC (A to C)” has been assigned as a tRF-5, because we failed to find its identical sequence in any database and it mapped to tRF5-GlyGCC perfectly, except that the third nucleotide was C while the correct one in the mouse genome database is A.

As seen in Figure 1A-B, there were several tRF isoforms that were of an identical 5'-end but of different 3'-ends. The major isoforms were 32-34 nts long (for tRF5-ValGTG) and 30-32 nts long (for tRF5-GlyGGY). ANG cleavage sites, estimated from the 3'-ends of the tRF isoforms, were located at the immediate 5’-side of the anticodon. Interestingly, we also captured smaller tRF5-ValGTG of 23 nts (Figure 1A). We designated it as “tRF5-ValGTG(s)” to distinguish from the major tRFs of 32-34 nts which were designated as “tRF5-ValGTG(l)”. It should be noted that some previous studies detected tRF-5s of 30-35 nts [26,27,38], while other previous studies detected tRFs of ~22 nts [32,37].

The expression of tRFs was confirmed by a conventional technique. We chose Northern hybridization as the most suitable method to confirm a tRF, because this assay visualizes a discrete band for the presence of a specific tRF. We detected tRF5-ValGTG(l) (Figure 1C) and tRF5-GlyGGY(l) (Figure 1D) at sizes consistent with our deep sequencing data, as well as their mature tRNAs at ~75 nts. Also, tRF5-ValGTG(s) was clearly visible at ~23 nts (Figure 1C). All these bands were prominent and discrete along each lane (Figure 1C-D), reinforcing that tRFs were specific cleavage products. The expression level of tRFs appeared to be very abundant, especially when their band intensities were compared to those of highly abundant mature tRNA. Most importantly, the expression of tRFs was elevated upon infection by rickettsiae.

The data presented above were from mouse tissues. We next questioned if the tRNA cleavage is also seen in human cells. Sequence comparisons revealed that these two tRFs are conserved between mice and humans. Indeed, we could also detect these two tRFs in human endothelial cells (Figure 1E-F). tRFs were barely detected in mock-infected HUVECs (lane 4 in each panel), but became evident after the cells were infected with SFG rickettsiae (lane 3 in each panel). The tRF bands were pronounced after treating with exogenous rANG in addition to infection of the cells with SFG rickettsiae (lanes 1-2 in each panel). Collectively, our data demonstrate tRNA cleavage by ANG in both mouse and human cells infected with SFG rickettsiae.

Recent studies have indicated that some tRFs regulate their target mRNAs, like microRNAs [37]. Several studies suggested that a subset of tRFs share a similar mechanism to microRNA that recognize complementary sites in the 3'-untranslated regions of target mRNAs and repress gene expression, while another subset of them have a different mechanism [36,41]. For example, a 31-nts long tRF-5 has been shown to inhibit target mRNA provided with a complementary target site [29]; however, this trans-silencing activity appeared to be distinct from that of microRNAs. tRFs of our interest were 30-34 nts long (Figure 1A-B), and significantly longer than an average microRNA size. Thus, we reasoned that our tRFs will also act differently from microRNAs but similarly to the 31-nts long tRF-5.

To find potential targets, we searched for mRNAs with a sequence homologous to tRF5-ValGTG and tRF5-GlyGCC by using the BLAST algorithm. We did not use well-established tools for microRNA target prediction, because they give too much weight to the first eight nucleotides of a sncRNA (called the “seed sequence”). As aforementioned, our tRFs[36,41] are not thought to act through a microRNA mechanism. The BLAST outputs were further subjected to the RNAhybrid program (http://bibiserv.techfak.uni-bielefeld.de/rnahybrid/) to evaluate their base-pairing interaction with tRF-5. Based on ∆ G values, interactors of significant stability (-30 kcal/mol as a cutoff) were selected and tabulated in Table 3. Among them, PRKCB, SH3GLB1, and SNTB1 were of particular interest because they were determined to be relevant to diverse cellular signaling pathways, including endothelial cell proliferation and barrier function, nitric oxide synthase (iNOS and eNOS), and regulation of autophagy and apoptosis [46-48].

**Table 3:** The predicted interactors to the two tRF-5s.

**tRF**	**Gene name [accession number]**	**Base-paring to potential target sites**	**ΔG value (Kcal/mol)**
**tRF5-ValGTG**	Homo sapiens protein kinase C, beta (PRKCB), transcript variant 1, mRNA [NM_121535.2]		-34.2
	Homo sapiens carboxyl ester lipase (bile salt-stimulated lipase) (CEL), mRNA [NM_001807.3]		-35.3
	HOMO sapiens glutamyl-tRNA synthetase 2, mitochondrial (EARS2), transcript variant 1, mRNA [NM_001083614.1]		-40.2
	HOMO sapiens solute carrier family 23 (nucleobase trnasporters), member 2 (SLC23A2), trnascript variant 1, mRNA [NM_005116.5]		-36.9
**tRF5-GlyGCC**	Homo sapiens long interegenic non-protein coding RNA 317 (LINC00317), non-coding RNA [NR_038872.1]		-50.6
	Homo sapiens SH3-domain GRB2-like endophilin B1 (SH3GLB1), transcript variant 1, mRNA [NM_016009.4]		-37.3
	Homo sapiens glial cells missing homolog 1 (drosophila) (GMC1), mRNA [NM_016009.4]		-37.2
	Homo sapiens syntrophi, beta 1 (dystrophin-asociated protein A1, 59kDa, basic component 1) (SNTB1) mRNA [NM_021021.3		-39.7

The potential target mRNAs of the two tRF-5s and their interactions are summarized and tabulated. In the depicted base-pairing interactions, the sequences on the top and bottom are tRF-5 and its predicted target mRNA, respectively. The numbers of target mRNAs indicate nucleotide coordinates of the RefSeq shown in the second column. The degree of interaction, expressed in ∆ G values, was calculated by the RNAhybrid program and shown.

## **Discussion and conclusions**

A major clinical hallmark of SFG rickettsial disease is the infection of ECs leading to enhanced vascular permeability [8]. In the present study using a HUVEC model, we confirmed previous observations from different *in vitro* endothelial cell models [49-51] that endothelial permeability was enhanced upon infection endothelial cells with SFG *R. conorii*. However, the cellular and molecular mechanisms by which SFG rickettsiae increase endothelial permeability remain to be elucidated. Our previous investigation [11] demonstrated that tyrosine phosphorylation of endothelial adherens junctional protein VE-cadherins involved alterations in calcium-dependent homophilic protein-protein adhesion forces between VE-cadherins on adjacent cells, underlying endothelial paracellular dysfunction following SFG rickettsial infection. Therefore, phosphorylation of transmembrane adhesion proteins may serve as a pivotal regulator in endothelial barrier dysfunction[11]. Yet the implied direct association and the pathway to link SFG rickettsial infection of the endothelium and VE-cadherin phosphorylation remain unproven.

There are many mechanisms that regulate VE-cadherin stability at adherens junctions through modulating phosphorylation that effectively control VE-cadherin availability at the endothelial surface [10,15]. Angiogenic stimuli such as vascular endothelial growth factor (VEGF) [15], epidermal growth factor (EGF) [14], fibroblast growth factor (FGF) [12], and angiopoietin-1 [16] have produced inconsistent effects on induction of tyrosine phosphorylation of VE-cadherin. The literature suggests that VEGF and EGF may trigger tyrosine phosphorylation of VE-cadherin, disassembling the adherens junction complex through Src and Rac pathways [14,15]; angiopoietin-1 and FGF may dephosphorylate VE-cadherin to stabilize the endothelial barrier apparatus associated with β-integrin [12,16]. Observations from the present study displayed that ANG, an early identified angiogenic factor, was remarkably increased in endothelial layers in the microvasculature in multiple organs post-infection with *R. conorii*, and co-localized with SFG rickettsiae in the lesion. In liver, greater accumulation of ANG located at the endothelial layer of hepatic sinusoidal blood vessels compared to hepatocytes (Figure 2H) suggest hepatocytes as potential sources of increased synthesis of ANG after SFG rickettsial infection, but endothelial cells are the targets of upregulated ANG.

ANG was originally isolated from conditioned media of human colon adenocarcinoma cells based on its angiogenic activity [52]. To induce a wide range of cellular responses, ANG must undergo nuclear translocation and enhance rRNA transcription, the rate-limiting step of ribosome biogenesis and cell growth [21,53]. However, *in vitro* studies showed that confluent quiescent endothelial cells do not internalize exogenous ANG [21]. In the present study, rickettsial infection sensitized confluent HUVECs and initiated translocation of exogenous ANG in a compartmentalized pattern at different times post-infection (Figure 3). Typically, at 24 hrs post-infection, exogenous ANGs were in the nuclei. In contrast, at 72 hrs p.i. translocation of ANG predominated in cytoplasm. The underlying mechanisms associated with compartmentalized translocation during different intervals post-infection by SFG rickettsiae still needs to be investigated. Current evidence suggests that nuclear translocation of ANG is via an unidentified receptor(s) system and independent of lysosomes and the microtubule system [18,25], but is strictly dependent on endothelial cell density [21]. These conditions imply the probability that suppressive signaling from confluent quiescent endothelial cell–cell adhesion blocks endocytosis of ANG, or activation of an unknown ANG-specific transporting system by adverse environments (e.g., stress or intracellular pathogen invasion) initiates internalization of ANG. These two may function as an off–on switch.

Recent studies on the biological activity of ANG have been extended from enabling cell growth and proliferation to sustaining endothelial cell survival under adverse conditions [26-29]. Such emerging roles of ANG are distinct from those of nuclear located ANG. Nuclear translocation in growing endothelial cells and malignant cells correlates with cell proliferation through promoting rRNA transcription and protein synthesis [20,21]. The role of ANG under stress is envisioned to be activated in the cytoplasm and to mediate reprogramming of global protein translation, saving anabolic energy, and promoting cell survival during adverse events [18]. Our data directly demonstrated that SFG rickettsial infection of endothelial cells induced dynamic translocation of exogenous ANG into different endothelial compartments at different times post-infection. During early infection, nuclear translocation of ANG was not associated with functional evidence of increased endothelial permeability. The illustrated translocation of ANG in cytoplasmic compartments at 72 hrs p.i. (Figure 3) was concomitant with increased endothelial permeability, which was moderated by an ANG translocation inhibitor (Figure 4). Given the facts that phosphorylation of VE-cadherin was enhanced and endocytosis of disassembled VE-cadherin increased when cytoplasmic translocation of exogenous ANG took place, we conclude that, in addition to its angiogenic role, ANG is a key enhancing factor on endothelial barrier dysfunction. The mechanism by which SFG rickettsial infection-triggered cytoplasmic translocation of ANG results in phosphorylation of VE-cadherin mediated endothelial barrier dysfunction has yet to be determined.

ANG is known as the only angiogenic protein to specifically cleave tRNA *in vivo* and *in vitro*[18,42]. Although ANG protein consists of 123 amino acid residues [54], three distinct functional sites have been identified, including a receptor-binding site, a nuclear localization sequence, and a catalytic site executing ribonucleolytic activity [53,55,56]. Our *in vitro* assay demonstrated that ANG indeed cleaved human tRNAs to produce tRFs. Also, our studies of mouse tissues and HUVECs showed a clear correlation of cellular tRF-5 generation with the cytoplasmic localization of ANG, suggesting that tRNA cleavage by ANG also occurs *in vivo* when endothelial cells are infected with SFG rickettsiae. Our data showed that the major cleavage products were tRF-5s longer than 30 nts, and these tRF-5s were similar in length to the previously identified tRNA halves produced by a specific cleavage at the anti-codon loop [26].

In addition, we detected smaller tRF-5s of ~23 nts (for example, tRF5-ValGTG(s) in Figure 1C). These have also been described in human cancer cell lines [36,37]. One report showed that these are generated by Dicer, however, we do not know if this is also the case in the context of SFG rickettsial infection of endothelial cells. We examined microRNA expression profiles in our deep sequencing data, and failed to detect any global changes upon rickettsial infection, indicating that Dicer activity in general did not change. Also, it is not clear whether tRF5-ValGTG(s) is generated by ANG. In any case, these small tRF-5s were minor in quantity, relative to the longer tRFs, which we therefore pursued as a higher priority.

Our next study aim will be functional evaluation in the HUVEC model. We hypothesize that tRF-5s play a regulatory role by recognizing their target mRNAs in base-pairing interactions. This concept has been supported by recent experimental evidence [41]. We attempted to identify a target mRNA with functional significance. As shown in Table 3, our computational predictions indicated that tRF5-ValGTG and tRF5-GlyGCCtRFs have the potential to interact with transcripts associated with endothelial barrier function, host cell inflammatory response, and autophagy. These are direct topics to pursue in our future studies.

There were a number of limitations in our present study. First, use of neomycin could moderate the ability of exogenous ANGs to enhance endothelial permeability, yet there was elevated endothelial permeability compared to SFG rickettsial infection alone (Figure 4). To address this, future studies can include search for a specific noncellular-toxic ANG receptor inhibitor, other yet to be indentified pathways of tRFs biogenesis, and potential crosstalk between ANG-tRNA-derived sncRNAs and other pathways.

Second, regarding tRF profiling, although several tRFs were identified in the HUVEC model of SFG rickettsial infection and will be the subject of our next functional studies on HUVECs, we do not know the differences between primary human endothelial cells and endothelium in the mouse model. While the core components of the vertebrate microRNA pathway are highly conserved among species, the overall scenario of tRF is not well-understood. For long-term aims to study the functional role of tRFs in primary human endothelial cells, deep-sequence analysis and characterization of sncRNA in the HUVEC model is warranted.

Third and most importantly, the mechanism that regulates switching between the two distinct functional roles of ANG, promoting rRNA transcription in the nucleus and cleaving tRNA in the cytoplasm, is largely unexplored. The survival of mammalian cells exposed to stress requires a reprogramming of protein translation, which is regulated by a family of eukaryotic initiation factors (elF) [27]. Transfection of stress-induced tRFs could repress translation of mRNAs encoding “housekeeping” proteins and trigger the phospho-elF2α-independent stress granules that are essential components of the stress response [27,29]. Displacement of the elF4F complex is one mechanism by which stress-induced tRFs reprogram protein synthesis [29]. Data from the present study indicate that SFG rickettsial infection of endothelial cells could induce tRNA derived tRFs, and may protect endothelial cells post-infection. However, subsequently triggered endothelial barrier dysfunction causes secondary injury in tissues and organs, including edema and hypoxia. The underlying mechanisms that regulate this biopathologic outcome are largely unknown and require further study.

Analysis of the interactions among enriched tRFs and potential mRNA targets has provided putative mRNA candidates for future studies. There were three tRF-mRNA interactions that attracted our immediate and high priority attention for future studies. The first is the tRF5 interaction with mRNA encoding ValGTC-β protein kinase C (PRKCB). PRKCB has been reported to be involved in several different cellular functions, such as endothelial cell proliferation and barrier function, B-cell activation, and induction of apoptosis [47,57,58]. Potential inhibition of this mRNA by this tRF is quite possibly relevant to the pathogenic mechanism in SFG rickettsioses. The second is tRF5-GlyGCC interaction with mRNA that encodes for syntrophin. Syntrophins are adapter proteins that use multiple protein interaction domains to localize a variety of signaling proteins to specific intracellular locations. These include nitric oxide synthase (iNOS and eNOS) that is important for intracellular killing, kinases, ion channels, and water channels [46,59,60]. This tRF5-mRNA interaction is also a putative topic for further functional studies in regard to immunity to and pathogenesis of SFG rickettsiae. tRF5-GlyGCC is the third interaction with mRNA, and we identified a target which encodes for endophilin B1 ( also known as Bax-interactin factor 1)[48]. Endophilin B1 has been reported to be a key regulator in autophagy through the Bcl2-associated X (Bax) protein pathway [48,61,62]. Further research into the roles of tRFs could potentially increase our knowledge regarding the pathogenesis of SFG rickettsiae.

## Competing interests

The authors declare that they have no competing interests.

## Authors’ contributions

BG designed the study, performed experiments, supervised experiments in the BSL3 and ABL3 facilities, analyzed data, and wrote the manuscript. YL designed the study, performed experiments, analyzed data, and wrote the manuscript. IL performed Bioinformatics analysis. TRS, GX, and NLM. performed experiments in the BSL3 and ABSL3 facilities. NK, KL, SJ and BHJ. performed RNA relevant experiments. QC and TH performed histology and *in vitro* experiments. XC analyzed data and assisted in revision. DB assisted in directing the studies in the BSL3 facility. PJB analyzed pathological samples. TGK assisted in designed BSL3 experiments. DHW assisted in the design and analysis of studies, directed studies in the BSL3 and ABSL3 facilities, and wrote the manuscript. All authors read and approved the final manuscript.

## Pre-publication history

The pre-publication history for this paper can be accessed here:

http://www.biomedcentral.com/1471-2334/13/285/prepub

## Supplementary Material

Additional file 1 ***in vitro *tRNA cleavage by ANG.** 15% denaturing polyacrylamide gel showing *in vitro* cleavage of tRNA by rANG (recombinant human ANG). Mature tRNA and its cleavage products are indicated. Click here for file
