# Supplementary figures and images for: Compartmentalized, functional role of angiogenin during spotted fever group rickettsia-induced endothelial barrier dysfunction: evidence of possible mediation by host tRNA-derived small noncoding RNAs
Source: BMC Infect Dis. 2013 Jun 23;13:285. doi: 10.1186/1471-2334-13-285 (PMC3699377; doi:10.1186/1471-2334-13-285)

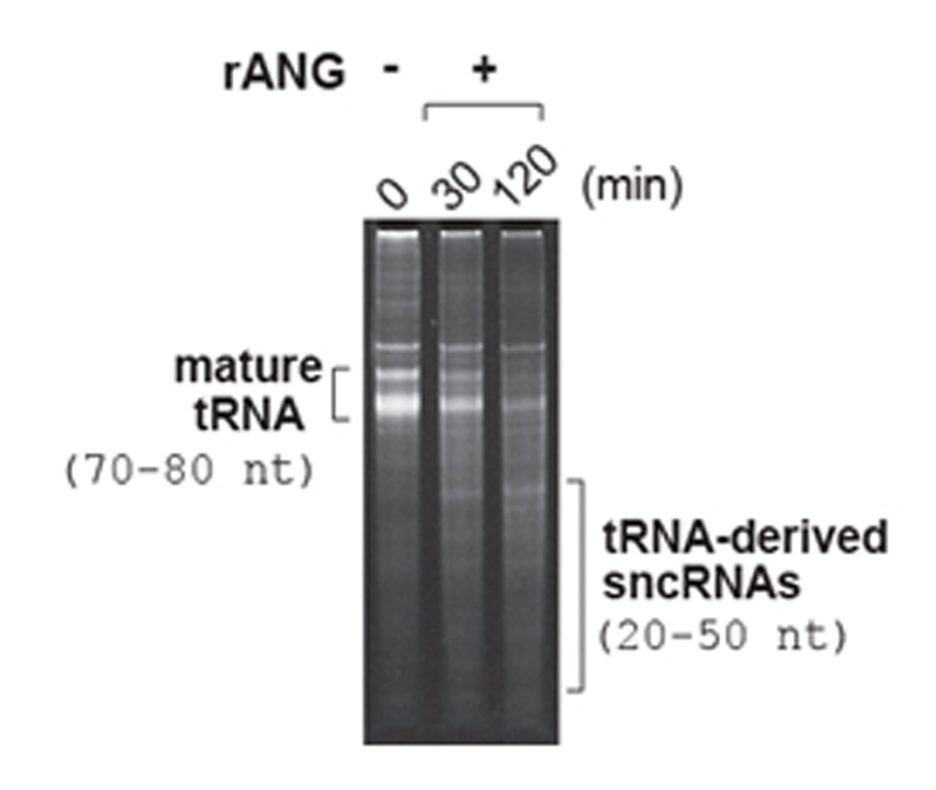

Supplement: Additional file 1 — in vitro tRNA cleavage by ANG. 15% denaturing polyacrylamide gel showing in vitro cleavage of tRNA by rANG (recombinant human ANG). Mature tRNA and its cleavage products are indicated. [file 1471-2334-13-285-S1.jpeg]
